# Family Perception and 6-Month Symptomatic and Functioning Outcomes in Young Adolescents at Clinical High Risk for Psychosis in a General Population in China

**DOI:** 10.1371/journal.pone.0138361

**Published:** 2015-09-22

**Authors:** Lu Wang, JingYu Shi, FaZhan Chen, YuHong Yao, ChenYu Zhan, XiaoWen Yin, XiaoYan Fang, HaoJie Wang, JiaBei Yuan, XuDong Zhao

**Affiliations:** 1 Department of Psychosomatic Medicine, Shanghai East Hospital, Tongji University School of Medicine, Shanghai, China; 2 Faculty of Humanities and Behavior Medicine, Tongji University School of Medicine, Shanghai, China; 3 Department of Psychosomatic Medicine, Tongji Hospital, Tongji University School of Medicine, Shanghai, China; 4 Department of Tongji university counseling center, Tongji University, Shanghai, China; Chiba University Center for Forensic Mental Health, JAPAN

## Abstract

**Background and Aims:**

Given the difficulty of treating schizophrenia and other forms of psychosis, researchers have shifted focus to early detection and intervention of individuals at clinical high risk (CHR) for psychosis. Previous studies have shown that elements in family functioning could predict symptom outcome in CHR individuals. However, associations between self reported family functioning and symptom or functioning outcome of CHR individuals was rarely reported. Our study aimed to investigate the characteristics and the role of family functioning in the development of CHR individuals among young adolescents.

**Methods:**

A sample of 32 CHR individuals was recruited from 2800 university students. The characteristics of family perception were evaluated by both Family Assessment Device (FAD) and Family cohesion and adaptability evaluation Scale II (FACES II). 6 month follow up data was available with 25 of the recruited CHR individuals. Baseline socio-demographic characteristics and family functioning were compared between CHR and control group. We also measured the associations between different dimensions of perceived family functioning and both severity of prodromal symptoms and global functioning at baseline and 6-month follow up.

**Results:**

CHR individuals showed more maladaptive family functioning compared to control in nearly all of the dimensions of FAD and FACES II except for Affective Involvement. Better Problem Solving and Affective Responsiveness predicted less severe positive and negative symptoms respectively. Family cohesion and adaptability were not only correlated with the baseline severity of general symptoms, but also positively associated with the general and disorganized symptom outcome.

**Conclusions:**

This study contributed preliminary evidence towards the associations between family perception and symptom outcome of CHR individuals. It also provided evidence for the importance of family interventions on CHR individuals.

## Introduction

In spite of advances in the understanding of their causes and treatment, schizophrenia and other forms of psychosis continue to be seriously disabling disorders. In recent years, the importance of early detection and prevention aiming at delaying or preventing individuals at clinical high risk (CHR) for psychosis from developing into fully psychotic symptoms attracted a lot of attention. Recently established methods for detection of CHR individuals allowed for investigation of factors protecting them from conversion to psychosis. Although genetic predisposition is a strong factor in the developing of psychotic disorders, the etiological model of schizophrenia were thought to be interacted by both vulnerability factors and psychosocial stress [1]. Psychosocial stress is a precipitating factor for individuals with a genetic diathesis. Evidence from adoption studies, expressed emotion has shown that family environment has substantial impact on the development of psychosis. One recent adoption study showed that the adoptive family rearing environment can be predictive of schizophrenia spectrum disorders [2]. High levels of expressed emotion in a family can be a risk factor for the relapse of psychotic disorders [1, 3, 4].

With the development of detection criteria of CHR individuals with prodromal syndromes, some researchers have been trying to investigate the role of family environment in the course of syndrome progression. O’Brien and its colleagues indicated that caregivers’ positive remarks and warmth were associated with improvement in negative symptoms and social functioning among CHR individuals at follow up [5]. They also showed that adolescents’ baseline constructive communication among family members was associated with enhanced social functioning, whereas the baseline conflictual communication was positively associated with increase in positive symptoms 6 months later [6]. Their studies have shown that there was a link between family environment and the development of CHR individuals with prodromal syndromes. However, studies investigating self-reported family functioning in CHR adolescents are very limited. Observer-based measures may not be as predictive as members’ perceptions of their family environment [7, 8]. Previous studies have also shown that patient perceive family functioning differently than their family members do. Thus it is important to understand the role of self reported family functioning in the course of CHR individuals. Only one previous study investigated the characteristics of family perception in CHR individuals. It showed that CHR individuals were likely to report higher levels of perceived family dysfunction compared to a community sample of young people [9]. However, it is inappropriate to use a community control as their participants are from clinical sample, and the data in this study was cross-sectional, which limits the ability to infer causality between these constructs. To our knowledge, the longitudinal role of family functioning in the development of CHR individuals, especially in a non-clinical population, has not been investigated yet. CHR studies, to date, focused almost exclusively on help-seeking population without venturing into the general population. However, it is important to understand the prodromal syndromes in the general population to have a complete understanding of it. Family functioning was indicated to be a protective factor in help-seeking CHR individuals, but in the non-clinical population, it has not yet been investigated. The aim of the present study was to investigate the characteristics of self-reported family functioning in CHR individuals in a general population and subsequently the role of family functioning in the course of CHR individuals.

## Methods

### Participants

The study protocol and informed consent procedures were approved by Tongji University in Shanghai, China. Written informed consent was obtained form all participants. We used a structured interview for Prodromal Syndromes (SIPS [10]) to determine whether they met the criteria of CHR only with those scored higher than 6 in PQ-16. Exclusion criteria include a DSM-IV diagnosis of mental retardation, schizophrenia or schizoaffective disorder, past or present psychotic episodes, current drug or alcohol dependence, and/or the presence of a neurological disorder. Among all the screened students, there were 32 participants who met the research diagnosis criteria for Prodromal syndrome on the SIPS during the consecutively detection period from September 2013 to March 2014. All participants (n = 32) were first or second year undergraduate students, and were unmarried. The mean age of the entire sample was 18.8 years (SD = 1.07 years), 13 of them were female.

### Procedure

**Fig 1:**
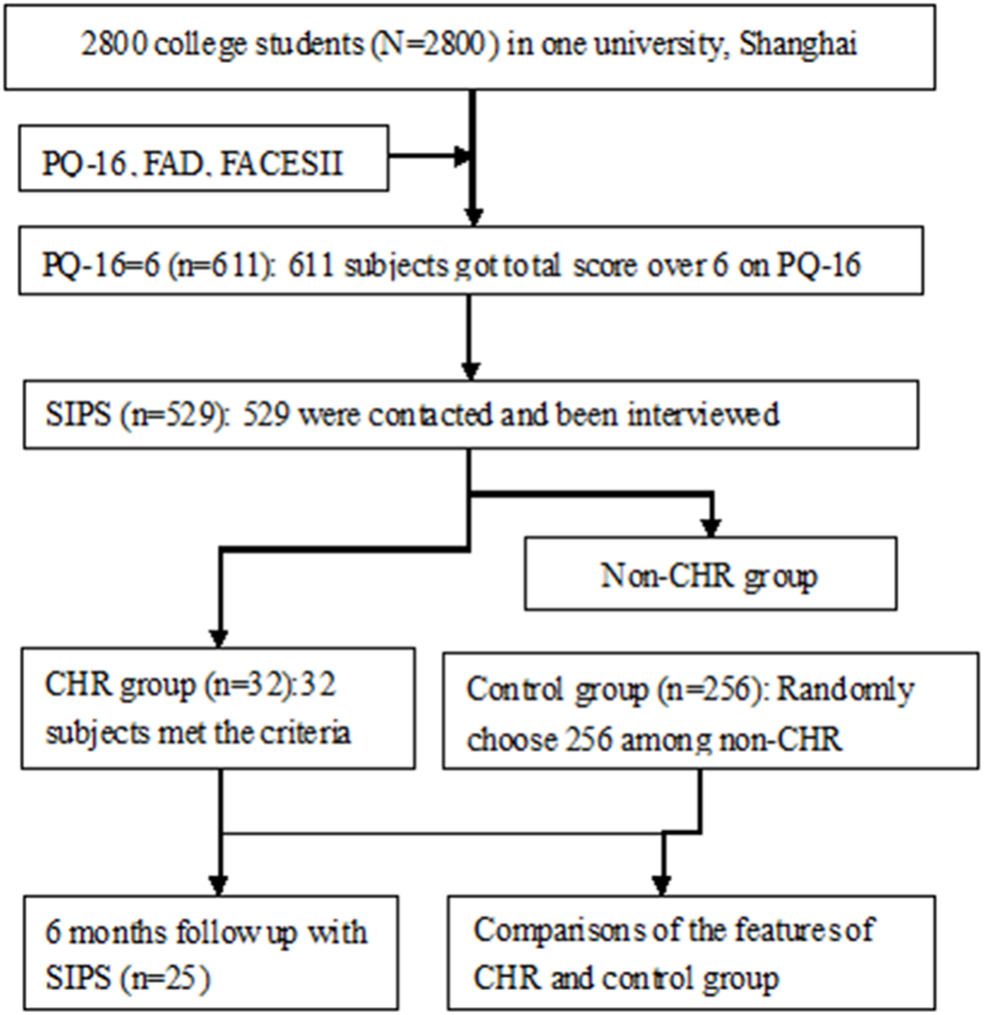
Flowchart of the study. The flowchart has been presented below illustrating the procedure of this study.

Among all of the 2800 participants who returned the questionnaires, 2336 completed all the questionnaires including CPQ-16, FAD and FACESII-CV.We conducted SIPS interviews with those who scored higher than cut off score 6 on CPQ-16. Among all the respondents, 611 students achieved a score of 6, wecontacted529 of them, all of them were interviewed by psychiatrists who underwent a standard training workshop. Finally, 32 of them met the criteria of CHR on SIPS. Because gender can make a difference on perceived family functioning, we used a random number generator to randomly select 104 females and 132 males from the 497 SIPS negative individuals as the control group. This matched the gender ration of the CHR group. 6 month follow up clinical and functional outcome data were available for 25 of the included 32 CHR adolescents. There were no pharmaceutical or psychological interventions during this period. At the end of 6 month follow up, 2 individuals converted to psychosis, one converted to bipolar disorder, one converted to schizophrenia. From the 7 persons who refused to come for a follow up interview at 6 month, 6 reported during a telephone conversation that either their symptoms have got better or those symptoms even no longer existed. The one remaining person could not be contacted. The flowchart of this study is presented below (Fig 1).

### Instruments

#### The 16-Item Version of the Prodromal Questionnaire (PQ-16)

PQ-16 is a self-reported questionnaire to screen individuals with psychosis risk [11], it only takes a few minutes to complete. Items were answered true or false according to the individuals’ experiences in the last month; if the answer was false, the item was rated 0, and if true, the distress scores were rated according to 4 degrees ranging from 0 (no) to 3 (severe). The total score was the sum of all entries scores. One previous study of CPQ-16 (Chinese version of PQ-16) in China demonstrated that a cut off score of 9 would have the greatest sensitivity (85%) and specificity (87%) [12]. In our study we use a cut off score of 6 to decrease the rate of missed diagnosis, according to Ising and its colleagues [11].

#### McMaster Family Assessment Device (FAD)

The FAD is a 60-item self reported scale according to the McMaster Model of Family Functioning (MMFF) [13] that measures the perceptions of participants in seven dimensions of family functioning: General Functioning, Problem Solving, Communication, Roles, Affective Responsiveness, Affective Involvement and Behavior Control. Each of the items is scored on a 1–4 Likert format scale, the higher score indicates the worse family functioning. The FAD has been translated into Chinese (in Hong Kong), the validity and reliability of the Chinese version of the FAD has been investigated in Hong Kong [14, 15]: the internal consistency of the all the dimensions is fair to excellent with coefficient alphas ranging from 0.53 to 0.94 and the test-retest reliability is 0.53 to 0.81.

#### The Family Adaptability and Cohesion Evaluation Scale, second edition, Chinese version (FACESII-CV)

FACES II [16] is a 30-item self-report scale that evaluates two dimensions of family life: family cohesion and family adaptability. Each of the items is scored on a 1–5 likert format scale with higher score indicating better family functioning. The FACES II has been translated into Chinese (FACESII*-*CV) in 1992 and shows both a good internal consistency (0.73~0.85) and test-retest reliability (0.84~0.91). And in the study of Phillips [17], FACESII-CV also shows a good psychometric properties. Usually, respondents are asked to answer the questions twice, first to evaluate subjects’ perception of actual family cohesion and adaptability, second to evaluate their perception of ideal family conditions. In this study, we only measures the actual family cohesion and adaptability as we only wanted to know the characteristics of family functioning among participants.

FAD and FACES II evaluate different aspects of family functioning, as they originated from different theory systems. To better understand the characteristics of family functioning among CHR individuals, both FAD and FACES II were used to assess perceived family functioning of CHR individuals in our study.

#### The Structured Interview for Prodromal Syndromes (SIPS)

SIPS [18] is a semi-structured interview designed to set the criteria of recruiting people with CHR and be administered by trained evaluator. On the Scale of Prodromal Symptoms (SOPS) [19] there are four various dimensions; It comprises the assessments of 5 positive symptoms, 6 negative symptoms, 4 disorganized symptoms and 4 general symptoms. Those who met any one of the following three syndromes would be diagnosed with CHR: 1. Attenuated Positive Symptom Prodromal Syndrome (APSS); 2. Brief Intermittent Psychosis Prodromal Syndrome (BIPS); 3. Genetic Risk and Deterioration Prodromal Syndrome (GRDS). The SIPS also includes the assessment of functioning by the Global Functioning Scale(GAF-M) [20]. The Chinese Version of SIPS has been shown to have good reliability and validity in the assessment of CHR[21]. We used the summed scores of positive, negative, disorganized and general symptoms in our analysis.

All screening staff members have completed a SIPS training workshop. All Screening staff achieved high inter-rater agreement (0.91–0.98) on intra-class correlations of SOPS items and perfect agreement for SIPS diagnoses (κ = 0.97) on 3 SIPS training videotapes.

#### Sociodemographic characteristics

Social demographic characteristics were evaluated by a demographic questionnaire, designed by the researchers, including name, gender, age, discipline, grade, marriage status, single child or not, relationship between parents, being raised by others in their childhood, family history of psychosis, family income.

### Statistical analysis

The socio-demographic and family functioning features were described using mean values with standard deviations or frequencies with percentages according to the nature of the variable. We examined for normality and homogeneity of various variables. For the comparisons of socio-demographic characteristic, independent student’s t-test was used when normality assumption held, chi-square tests was employed for categorical variables and a chi-square trend was used for ordinal data. Independent student’s t-test was also used for the comparisons of family functioning measured by FAD and FACES-II between CHR and control. In order to investigate the relationship between baseline family functioning and severity of symptoms and global functioning, Pearson’s correlations were calculated. Relationship between family perception and symptom severity and global functioning were assessed by partial correlation test controlling for baseline severity of symptoms.

## Results

### Sociodemographic characteristics of CHR individuals

Among 2800 returned questionnaires, 464 were invalid because more than 1 percent of questions were umcompleted. Finally, 32 (1.4%) met the criteria of CHR. The average age of included CHR individuals was 18.78, ranging from 17 to 21. All of them were unmarried. A majority of them were from single child families of Han ethnicity. All of them were either freshmen or sophomores in college. Among total subjects of 32, 19 of them were males, slightly over represented than female, which is in line with the gender distribution in psychosis (Table 1). All of the 32 CHR individuals met the criteria of APSS, 7 of them also met the criteria of GRDS, while none of them met the criteria of BIPS.

**Table 1:** Characterizations of study participants and comparisons between CHR and control group.

Demographic information	CHR group	Control group	Statistic value	df	Pvalue
Age (mean±SD)	18.78±1.07	18.95±1.014	t = 0.862	286	0.390
Gender, number (%)			χ^2^ = 0	1	1
Female	13 (40.6)	104 (40.6)			
Male	19(59.4)	152 (59.4)			
Grade, number (%)			χ^2^ = 0.33	1	0.569
Fresh	25(78)	188 (73.4)			
Sophomore	7(22)	68 (26.6)			
Ethnic, number (%)			χ^2^ = 0.79	1	0.67
Han	29(90.6)	237 (92.6)			
Others	3(9.4)	19 (7.4)			
Single child, number (%)					
Yes	26(81.3)	193(75.4)	χ^2^ = 0.54	1	0.464
No	6(18.8)	63(24.6)			
Relationship of parents, number (%)			Z = -3.078		0.002**
Harmony	17(53.1)	199 (77.73)			
Ordinary	8(25)	34 (13.28)			
Frequent quarrels	2(6.3)	7 (2.7)			
Separated or divorced	5(15.6)	16 (6.25)			
Raised by others (not parents) in their childhood, number (%)			χ^2^ = 0.95	1	0.330
Yes	13 (40.6)	82 (32)			
No	19(59.4)	174 (68)			
Family history of psychosis, number (%)			χ^2^ = 0.13	1	0.723
Yes	0 (0)	1 (0.03)			
No	31 (100)	255 (99.7)			
Family month income (RMB), number (%)			Z = -0.602		0.547
<2000	2 (6.3)	28 (10.94)			
2000–4999	10 (31.3)	73 (28.52)			
5000–9999	16 (50)	88 (34.37)			
10000–19999	4 (12.5)	45 (17.58)			
>20000	0 (0)	22 (8.59)			

**p<0.01. CHR group = Clinical high risk for psychosis group

Comparing to the control group, which is chosen from the SIPS negative group, no significant differences were found in average age, gender, grade and ethnicity. CHR individuals reported worse relationship between their parents compared to the control group.

### Comparisons of family functioning between CHR and control group

**Fig 2:**
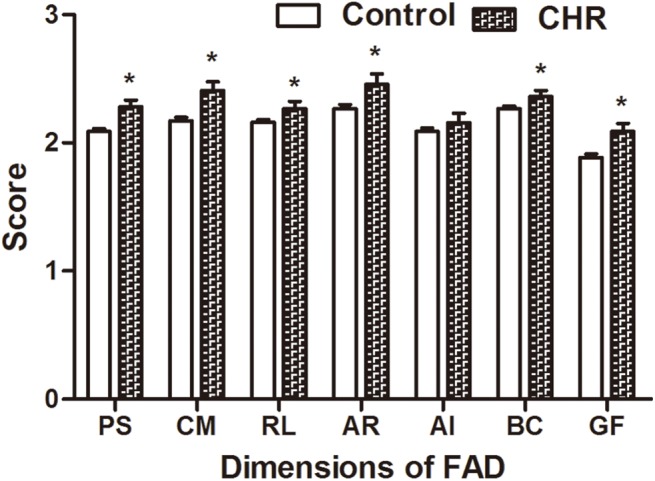
Comparisons of family functioning assessed by Family Assessment Device (FAD) between CHR and control group. This figure shows the difference between CHR and control in various dimensions of the Family Assessment Device. CHR means Clinical high risk for psychosis; FAD = Family Assessment Device; PS = Problem Solving; CM = Communication; RL = Roles; AR = Affective responsiveness; AI = Affective Involvement; BC = Behavior Control; GF = General Functioning. *P<0.05.

**Fig 3:**
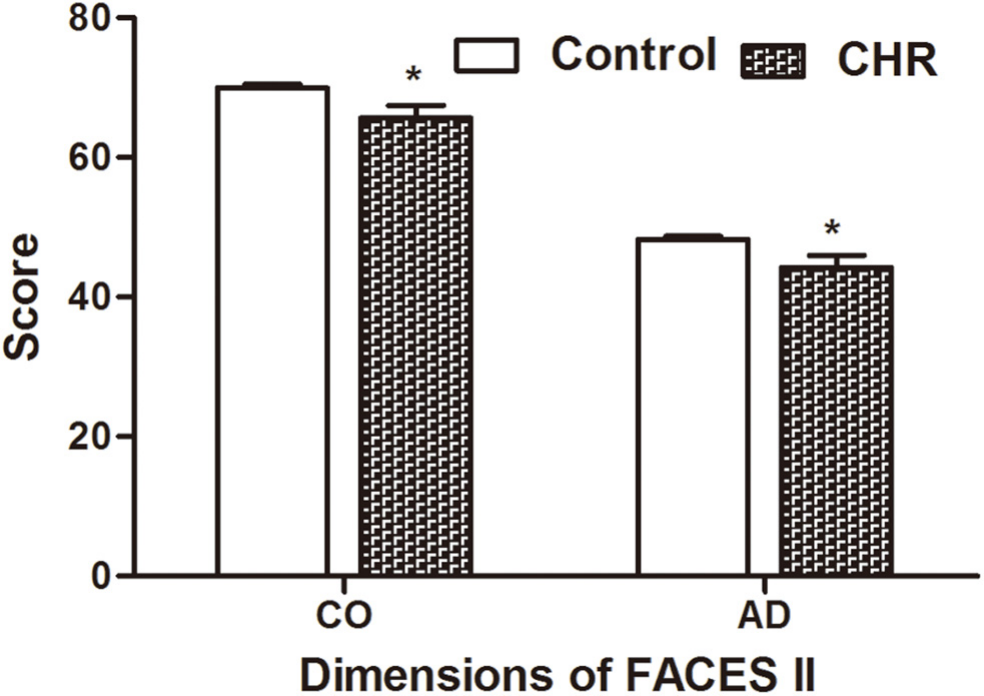
Comparisons of family functioning assessed by Family Cohesion and Adaptability Scale between CHR and control group. This figure shows the difference between CHR and control in level of cohesion and adaptability of family. CHR means Clinical high risk for psychosis; FACES II = Family adaptability and cohesion evaluation scale, second edition; CO = family cohesion; AD = family adaptability; GAF = functioning assessed by GAF-M. *P<0.05.

As presented in Fig 2, the CHR group showed worse family functioning than the control group in dimensions of Family Assessment Device (FAD) including: Problem Solving (p = 0.03), Communication (p = 0.002), Roles (p = 0.04), Affective Responsiveness(p = 0.025), Behavior Control (p = 0.47) and General Function (p = 0.006). They also scored less than the control group in both cohesion and adaptability in FACES II (Fig 3), which also indicated worse family functioning in the CHR group. The two groups achieved a similar score only only in the subscale of Affective Involvement (p = 0.19) in FAD.

### Associations between baseline family functioning and the baseline severity of symptom and global functioning

There were no correlations between any dimensions of FAD with either symptom severity or global functioning evaluated by GAF-M. Family cohesion and adaptability in FACES II were negatively related to the severity of the total scores of general symptoms, but no significant correlations were found between FACES II and other symptom subscales of SIPS including positive, negative, disorganized symptoms (Table 2). However, none of these correlations remained significant after corrections for multiple comparisons.

**Table 2:** Pearson correlations between FACES II and symptomatology and global functioning.

Variable		SOPS symptoms			GAF
	Positive	Negative	Disorganized	General	
CO	-0.207	-0.03	0.111	-0.362*	-0.118
AD	-0.17	-0.023	0.183	-0.361*	-0.16

* p<0.05. CO = family cohesion; AD = family adaptability; GAF = functioning assessed by GAF-M.

### Associations between baseline family functioning and 6-month symptomatic and global functioning outcome

Table 3 shows the relationship of baseline family functioning including all dimensions of FAD and FACES II with symptoms and functioning outcomes 6 months later. Partial correlations were employed using the symptom and functioning score at 6 months follow up as dependent variable and their counterpart at baseline as controlling variable. The results showed that the score of Problem Solving was positively related with the severity of positive symptoms, and the score of Affective Responsiveness was positively correlated with the outcome severity scores of negative symptoms 6 months later. In terms of dimensions on FACES II, cohesion and adaptability were both negatively correlated with the score of disorganized symptoms and general symptoms at the 6 month follow up. However, these correlations didn’t survive after corrections for multiple comparisons.

**Table 3:** Partial correlations between baseline family functioning and symptoms and functioning outcome.

Variable		SOPS symptoms			GAF
	Positive	Negative	Disorganized	General	
FAD					
PS	0.345*	-0.175	0.164	-0.328	-0.146
CM	0.192	0.039	0.243	-0.264	0.046
RL	0.094	-0.19	0.043	-0.197	-0.139
AR	0.06	-0.38*	0.185	-0.327	0.041
AI	0.025	-0.079	-0.119	-0.212	-0.199
BC	-0.062	-0.134	-0.076	0.06	-0.25
GF	0.096	-0.045	0.189	-0.389	0.073
FACES II					
CO	-0.299	-0.041	-0.57**	-0.377*	0.232
AD	-0.252	-0.002	-0.582**	-0.404*	0.094

* p<0.05

** p<0.01. FAD = Family Assessment Device. FACES II = Family adaptability and cohesion scale, second edition. PS = Problem Solving; CM = Communication; RL = Roles; AR = Affective responsiveness; AI = Affective Involvement; BC = Behavior Control; GF = General Functioning. CO = family cohesion; AD = family adaptability; GAF = functioning assessed by GAF-M.

## Discussion

This is the first study to use FAD and FACES II to assess family functioning of individuals at risk of psychosis in college students in China. This is also the first exploratory study that tested the longitudinal effects of self-perceived family functioning on the outcomes of symptoms and functioning among CHR individuals.

In this study, the incidence rate of CHR in college students was 1.4%, which is lower than 4% in the help-seeking population[22]. The reason for this difference might be that help seeking population already have troubling mental symptoms, it is possible that this population have higher risk for psychosis. Among 32 of the included participants, all met the criteria of APSS, seven met GRDS and none met BIPS, this finding was similar with findings in other studies. One study in North America showed that 96% of included high risk individuals met the criteria of APSS, less met GRDS or BIPS [23].

No significant group differences in demographic characteristics were found between CHR and control except for the parents’ relationship. Individuals with CHR were more likely to exist in families with bad p relationships between the parents; meanwhile, we identified that students who are at risk of psychosis perceived family functioning less favorably than didcontrol in nearly all FAD and FACES II subscales, except for the subscale of Affective Involvement (AI). These findings suggested that CHR individuals have more maladaptive family functioning than the control group. This is in line with the results of many other researches which demonstrated that individuals with psychosis reported low family functioning [24–26]. However, the result that the Affective Involvement, which reflects the family’s ability to care about and be interested in each other, is not different from control group was in contradiction with previous research which has demonstrated that individuals with psychosis have higher levels of expressed emotion(EE) in family, especially in the subscale of Emotion Over Involvement; Moreover, EE can be predictive of illness relapse in the long term [3, 27]. As we looked through the specific items of the Affective Involvement (AI), we found that only families where family members were extremely over interfering or extremely indifferent with each other would get high scores on this dimension, which means this subscale can only differentiate families with extreme dysfunction on affective involvement. However, it is highly possible that families of individuals with CHR, especially CHR individuals in general population only demonstrate relatively or mediate dysfunction on Affective Involvement, so that this subscale cannot identify the difference of AI between CHR and control group.

Another important finding was that family functioning assessed by FAD was associated with symptom outcome of CHR individuals although it is not related with the severity of symptoms at baseline. Baseline Problem Solving score was positively related to the severity score of positive symptoms 6 month later, which means those with better problem solving functioning have less severe positive symptoms after 6 months. This is very important because that positive symptoms signal the conversion from prodrome syndrome to an onset of psychosis. This result is similar from what O’Brien and her colleagues have found in a clinical sample [6]. They found out that the way how a family solves problems was associated with the symptomatic outcome, and positive symptoms improved in families solving problems via constructive communication rather than conflictual communication at 6-month follow up. Another subscale of FAD, Affective Responsiveness (AR) was associated with the severity of negative symptoms six months later. Affective Responsiveness (AR), as we have already mentioned in the method section, reflects whether family members experience and respond to the full spectrum of feelings experienced by human. Negative symptoms contain items such as social anhedonia, expression of emotion, experience of emotions and feelings. One explanation for this finding might be that if a young man grows up in a family where family members cannot experience or respond to others’ feelings and emotions, it is likely that they are not so capable of interacting well with others in a social situation and this could be the mechanism that counts for the association of AR and negative symptoms like social anhedonia over time. Those who are withdraw from social interaction, cannot test their thoughts in a relational context; this might increase their suspiciousness and delusional ideas. Although negative symptoms have not yet been used as criteria for the transition of CHR individuals, studies have shown that negative symptoms can predict subsequent transition to psychosis [28]. What’s more important is that most drugs for psychosis can only alleviate positive symptoms, with little effect on negative symptoms and social functioning [29, 30]. Considering the predictive effect of Affective Responsiveness among family members on the outcome of negative symptoms, psychosocial intervention especially family intervention might be effective on negative symptoms. One preliminary research has already demonstrated that family-focused treatment can improve negative symptoms in the long term [31]. What is more interesting in the Chinese context is that, it has a long tradition of Confucian culture, which focuses on practical order and rules with an especial emphasis of rules governing the family; it carries the notion that fathers guides sons and husbands guides wives. Confucian thoughts also attach high importance to sense and reason, neglecting feelings and emotions. Therefore we suppose that family interventions that focus on Affective Responsiveness might be even more important and effective in Chinese culture, however, further studies are needed to confirm this point.

In terms of findings on FACES II, compared to control group, CHR individuals perceived lower cohesion and poor adaptability, this is in line with what has been indicated in previous study in schizophrenia [17]. Baseline family cohesion and adaptability were correlated with baseline general symptom severity, moreover, at 6-month follow up, family with better cohesion and adaptability showed less severe scores in disorganized symptoms and general symptoms. These findings suggested that good emotional bonding and better adaptability to change might be protective factors for CHR individuals in the aspects of disorganized and general symptoms.

In the previous study, it has been shown that family functioning can predict better social functioning of individuals with psychosis [32]. O’Brien has also shown that constructive communication during the process of family problem solving was associated with better social functioning outcome [6]. However, in our study, there were no correlations between family environment and the outcome of global function of CHR individuals at the 6 month follow up. There were several reasons we consider in this contradiction. Foremost, the participants in our study were from a top rank university in China, they had good functioning at the beginning of this study, and after 6 months, many of them had improved in SOPS symptoms and sustained a stable global functioning, so there was less difference between baseline and 6 months follow up global functioning. Another plausible reason might be related to the small number of recruited participants, which made it difficult to detect the trends. Also the difference of family assessment instruments might also play a part in this.

Finally, it should be noted that, in this study, a large number of analyses were conducted on the same small sample, so that none of these correlations would survive after corrections for multiple comparisons. The results of this study should be considered exploratory and preliminary. However, as we screened a large number of college students, this may partially reflect the hard to reach nature of CHR, especially in the general population.Despite these limitations,our study is the first to investigate the relationship between perceived family functioning and CHR among the general population, this initial study provided a basis for further study of family functioning and family focused interventions on CHR individuals.Two previous researches have explored the effects of family intervention on CHR individuals. Family focused treatment on family relationship for young adolescents with CHR has been indicated to be effective on both positive and negative symptoms [31]. Another preliminary study that investigated the effect of group and family-based cognitive behavioral therapy program has shown relatively positive effects on symptom improvement [33]. As what was found in this study, family intervention with emphasis on family functioning, especially on problem solving, affective involvement, cohesion and adaptability might be also effective in improving symptom outcome in CHR individuals.

## Conclusions

This study provided preliminary evidence towards the associations between perceived family functioning and symptom outcome of CHR individuals. It indicated that family functioning could be predictors for the symptom outcome of individuals at clinical high risk for psychosis, thus it highlighted the importance of family interventions on CHR in the future.
